# MYIASIS IN A LARGE PERIGENITAL SEBORRHEIC KERATOSIS

**DOI:** 10.4103/0019-5154.70699

**Published:** 2010

**Authors:** Sudip Kumar Ghosh, Debabrata Bandyopadhyay, Sharmila Sarkar

**Affiliations:** *From the Department of Dermatology, Venereology, & Leprosy, R. G. Kar Medical College; 1 Khudiram Bose sarani, Kolkata-4, West Bengal, India. E-mail: dr_skghosh@yahoo.co.in*; 1 *From the Department of Psychiatry, Calcutta National Medical College, Kolkata 700014, India*

Sir,

Myiasis is not uncommon in rural areas and usually occurs in patients with precarious hygienic practices, psychiatric disturbances, diabetes, immunodeficiency, and low soceio- economic conditions.[1] This disease is common in tropical and developing countries and may often complicate preexisting dermatoses. We report a case of myiasis in a giant lesion of seborrheic keratosis on perineal and scrotal areas in a mentally disabled young man, for its rarity.

A 33-year-old, unmarried, newly diagnosed schizophrenic man presented to us with a large peri-genital lesion. He had a history of some asymptomatic, gradually progressing, darkly pigmented growth on the same region for the preceding five years. He had never sought any medical opinion for this condition previously. There was a history of ulceration of the lesions with expulsion of ‘worms’ for the preceding couple of weeks. He was not on any medication and had no other history of any systemic illness. There was no history of similar illness in the family. Although his mother accompanied the patient to our clinic, he lived completely alone in a rural area with evidence of social and family isolation.

**Figure 1:**
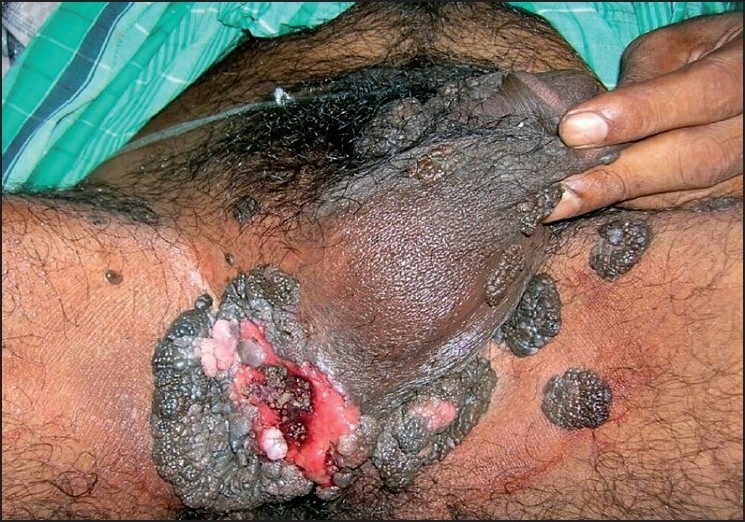
Multiple seborrheic keratoses with ulceration, focal necrosis, and larvae

**Figure 2:**
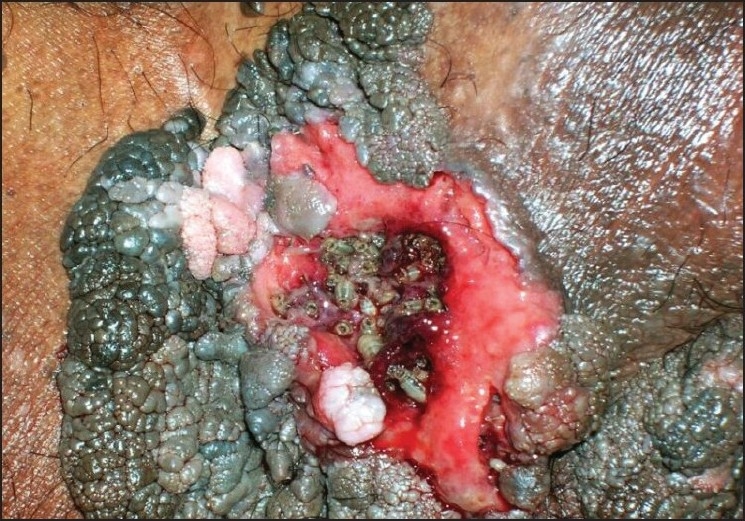
Close-up of larvae within an ulcerated, focally necrotic lesion of seborrheic keratoses

Examination revealed multiple verrucous, hyper pigmented, nodules and plaques of varying sizes ranging from 2 to 10 cm in diameter. The lesions had a stuck-on appearance and distributed on his perineal areas including the groins, scrotum and the penile shaft. The verrucous lesions were focally macerated, necrotic and whitish in appearance accompanied by ulceration with slight discharge of sero-sanguineous fluids. Multiple larvae were seen moving about inside the lesion. [Figures 1 and 2] There was no associated lymphadenopathy. The other areas of the skin were healthy and the cutaneous appendages were also normal. Systemic examination was noncontributory. Routine urine and stool examination was normal. Histopathological examination of multiple biopsy specimens revealed features typical of seborrheic keratosis. The larvae were manually extracted and later identified as *Chrysomyia bezziana*, based on the_characteristics of a mature larva and patterns of the anterior and posterior spiracles. The patient was prescribed systemic antibiotic to prevent secondary infection and was referred to plastic surgery department but was subsequently lost to follow-up.

Myiasis may occur with family abandonment as in this present case in which the myiasis as well as the large ulcerated perigenital lesion was grossly neglected. This disease usually affects the uncovered areas of the body, where the eggs are deposited more easily. There are two main types of myiasis, namely wound myiasis and furuncular myiasis, the later mimics a furuncle with a hole in the center containing a larva. Perineal myiasis in the form of involvement of a recto-cutaneous fistula and myiasis of the glans penis have rarely been reported. The male genital infestation is exceedingly rare, since the area is usually protected by clothes and is little accessible to the insects’ contact.[2] Our patient used to wear *‘lungi’* (a single piece of cloth worn around the lower part of the body) without any underwear for most part of the time leaving the peri-genital lesions vulnerable to infestation.

*Chrysomyia bezziana* infestation is common in tropical countries, especially in India. The larvae are photophobic and penetrate deep into tissue assisted by their sharp mouth hooks and anchoring inter segmental spines.[3] The larvae are identified by their characteristic morphology and microscopic appearance. Though a common cause of myiasis, its role in the causation of urogenital disease is extremely rare.[3] On the other hand, seborrheic keratoses are common benign skin tumors of the elderly occurring usually on the face, scalp, extremities, chest and back; but, its occurrence on genital and perianal locations is extremely rare.[4] Myiasis had been reported to secondarily affect giant squamous cell carcinoma, cutaneous melanoblastoma, Bowen’s disease or following radiotherapy of squamous cell carcinoma;[5] but its occurrence in a perigenital large seborrheic keratosis was an unusual feature in our case.
